# Evaluation of Hedgehog Pathway Inhibition on Nevoid Basal Cell Carcinoma Syndrome Fibroblasts and Basal Cell Carcinoma-Associated Fibroblasts: Are Vismodegib and Sonidegib Useful to Target Cancer-Prone Fibroblasts?

**DOI:** 10.3390/cancers13225858

**Published:** 2021-11-22

**Authors:** Laura Eibenschutz, Silvia Caputo, Emanuela Camera, Anna Carbone, Vitaliano Silipo, Emilia Migliano, Caterina Aurizi, Carlo Cota, Pasquale Frascione, Barbara Bellei

**Affiliations:** 1 Oncologic and Preventative Dermatology, San Gallicano Dermatological Institute, IRCCS, 00144 Rome, Italy; laura.eiibenschutz@ifo.gov.it (L.E.); anna.carbone@ifo.gov.it (A.C.); vitaliano.silipo@ifo.gov.it (V.S.); pasquale.frascione@ifo.gov.it (P.F.); 2 Laboratory of Cutaneous Physiopathology and Integrated Center of Metabolomics Research, San Gallicano Dermatological Institute, IRCCS, 00144 Rome, Italy; silvia.caputo@ifo.gov.it (S.C.); emanuela.camera@ifo.gov.it (E.C.); 3 Department of Plastic and Reconstructive Surgery, San Gallicano Dermatological Institute, IRCCS, 00144 Rome, Italy; emilia.migliano@ifo.gov.it; 4 Department of Dermatological Clinic, San Gallicano Dermatological Institute, IRCCS, 00144 Rome, Italy; caterina.aurizzi@ifo.gov.it; 5 Department of Dermophatology, San Gallicano Dermatological Institute, IRCCS, 00144 Rome, Italy; carlo.cota@ifo.gov.it

**Keywords:** basal cell carcinoma, skin, nevoid basal cell carcinoma syndrome, Gorlin syndrome, hedgehog pathway, CAF

## Abstract

**Simple Summary:**

Nevoid basal cell carcinoma syndrome (NBCCS) is a genetic disorder of autosomal dominant inheritance that dramatically predisposes a patient to the formation of basal cell carcinoma (BCC) due to causative mutations in several genes associated with the Hedgehog (Hh) pathway. Somatic mutations in this pathway are also associated with sporadic BCC, the most common form of skin cancer. Hh signalling extends its effect on tumorigenesis by modulating the tumour microenvironment in a paracrine fashion. Consistently, NBCCS fibroblasts could facilitate BCC occurrence. Here, we investigated vismodegib and sonidegib, two molecules currently used to target this pathway in cancer cells, as a therapeutic option against syndromic and BCC-associated fibroblasts.

**Abstract:**

Activating mutations in the Hh pathway underlies the development of sporadic and familial skin BCC. For these oncogenic proliferations displaying ligand-independent activation of the intracellular pathway, two molecules have been approved for therapeutic purposes: vismodegib and sonidegib. Improper Hh signalling occurs in many human tumours also via a paracrine mechanism (ligand-dependent) in which the secretion of Hh ligands by stromal cells support tumour growth. On the other hand, the mobilization of neoplastic stroma by cancer cells is sustained by the activation of Hh signalling in surrounding fibroblasts suggesting a central role of this bidirectional crosstalk in carcinogenesis. Additionally, loss-of-function mutations in the *PTCH1* gene in the context of NBCCS, an autosomal dominant disorder predisposing to multiple BCCs, determine tumour permissive phenotypes in dermal fibroblasts. Here, profiling syndromic and BCC-associated fibroblasts unveiled an extraordinary similarity characterized by overexpression of several Hh target genes and a marked pro-inflammatory outline. Both cell types exposed to Hh inhibitors displayed reversion of the tumour-prone phenotype. Under vismodegib and sonidegib treatment, the Wnt/β-catenin pathway, frequently over-active in tumour stroma, resulted down-regulated by pAKT-GSK3β axis and consequent increase of β-catenin turnover. Overall, this study demonstrated that vismodegib and sonidegib impacting on fibroblast tumour supportive functions might be considered in therapy for BCC independently to the mutation status of Hh components in neoplastic cells.

## 1. Introduction

Basal cell carcinoma (BCC) accounts for almost 80% of skin cancers [1,2] and its healthcare workload is principally within dermatology departments. Although most BCCs are small, well-defined tumours amenable to surgery or conservative procedures, in a small proportion of patients, BCCs can progress to an advanced stage including locally advanced BCC and metastatic BCC. Advanced BCC can cause disfigurement, morbidity, and lower a patient’s quality of life [3,4]. Thus, in these selected cases pharmacological therapies are preferable to surgical treatment. Non-surgical techniques include photodynamic therapy and topical imiquimod or 5-fluorouracil treatment whereas cytotoxic systemic chemotherapy has not been approved for the treatment of non-resectable BCCs. However, most of these approaches aim to obtain local control of slow-growing lesions, whereas advanced BCC can incompletely benefit from this non-resolutive care. Recently, vismodegib (GDC0449; trade name Erivedge) and sonidegib (LDE225; trade name Odomzo) two hedgehog pathway (Hh) inhibitors have been approved for oral treatment of patients with metastatic BCC or patients with recurrent, locally advanced BCC who are inappropriate for surgery and radiotherapy. The Hh signalling is a major regulator of cell differentiation, cell proliferation, and tissue polarity. The Hh pathway is largely inactive in adult tissue except for its function in tissue repair and regulation of adult stem cells but it is aberrantly active in several types of tumours [1,2,5]. In the canonical pathway, binding of Sonic Hedgehog (SHH), the most widely expressed hedgehog protein to its receptor, patched homolog 1 (PTCH1), releases inhibition on Smoothened (SMO), a 7-span transmembrane protein, decreasing the interaction between suppressor-of-fused homolog (SUFU) and the glioma-associated transcription factors (GLI1, GLI2, and GLI3), the terminal effectors of the pathway [6,7,8]. In mammals, Desert Hedgehog (DHH) and Indian Hedgehog (IHH) also function as ligands for PTCH1 resulting in intracellular signalling activation. The Hh target genes include proliferation and differentiation regulating genes (*CyclinD1* and *D2*, *E2F1*, *PDGFRα*, *IGFBP3*, and *IGFBP6*), cell survival regulating gene (*BCL-2*), pro-angiogenic factors (*VEGF*, *Cyr61*) as well as feedback genes of Hh pathway (*PTCH1* and *2*, *GLI1* and huntingtin interacting protein-1, *Hip1*) that further amplify signalling activation [8,9]. The Hh pathway is in conjunction with other important pathways, including epidermal growth factors (EGF/EGFR), transforming growth factor-β (TGF-β), Wnt/β-catenin signalling, PI3K/mTOR, NF-kappaB and Vitamin D [10,11,12]. Improper activation of Hh signalling is the hallmark of BCC pathogenesis [13,14]. The causative role of aberrant Hh signalling in BCC is underlined by the fact that approximatively 70–80%, 20%, and 8% have *PTCH1*, *SMO*, and *SUFU* driver mutations respectively [15,16,17]. Bi-allelic inactivating mutations are necessary for the loss of Hh pathway control that confers cellular growth deregulation, and, potentially, tumour development. Thus, pharmacological inhibition of the Hh pathway exerts an anti-tumor effect in several human cell lines, including renal carcinoma [18], oral squamous cell carcinoma [19], breast cancer cells [20] and skin basal cell carcinoma [21]. Another class of patients that benefices from Hh inhibitors are individuals with nevoid basal cell carcinoma syndrome (NBCCS) (also referred as nevoid BCC syndrome, Gorlin syndrome, or Gorlin Goltz syndrome, OMIM#109400), an autosomal dominant rare hereditary condition frequently due to germline mutation of *PTCH1* gene lacking significant genotype-phenotype correlations [22,23,24]. Prevalence of NBCCS is estimated from 1 per 57,000 to 1 per 164,000 with an even sex distribution and approximately 20–30% de novo mutation [25,26,27]. Affected individuals have two major phenotypes: developmental defects associated with generalized overgrowth and an increased risk of developing cancers including multiple BCCs (with a median age of onset of 20 years) frequently occurring in unexposed body areas [26,28]. In NBCCS patients presenting systemic loss of one functional copy (haploinsufficiency) of *PTCH1*, inactivation of the normal homolog by environmental mutagenesis or random genetic rearrangement confers ligand-independent activation of the Hh pathway and tumour permissive phenotype [29]. Unlike sporadic BCC patients, target therapy for these individuals necessitates a dual approach, focusing on the long-term preservation of healthy skin and reversing/limiting the growth of invasive tumours. A clinical trial including 41 NBCCS patients demonstrated that vismodegib significantly reduced the number of new BCCs per year and lowered the rate of surgically eligible tumour lesions, compared to the placebo arm [30]. However, the use of vismodegib in syndromic patients is limited by medication side effects and the consequent discontinuation rate. Thus, based on the idea that long-term therapy of syndromic patient needs a safer profile treatment, an alternative of topically applied sonidegib has been tested in a proof-of-concept clinical study confirming the therapeutic utility in BCC [31]. The response rate of syndromic patients to orally administered vismodegib is higher compared to sporadic probably due to the reduced mutational load of NBCCS-BCCs and the lack of intrinsic pre-existing resistance to Hh inhibitors [32,33,34,35]. Both vismodegib and sonidegib block hedgehog signalling by selective inhibition of SMO, even if chemical structures are different and they bind different residues of the same functional pocket eventually selecting distinctive mutation-based resistance [36,37,38]. Although the two principal patient-based studies, ERIVANCE (vismodegib) and BOLT (sonidegib), validated the therapeutic use demonstrating similar efficacy and patterns of adverse events, some studies evidenced relevant pharmacological differences [39,40]. One of the most interesting discrepancies resides in molecular distribution. Sonidegib may achieve a significantly higher concentration in the skin and other tissue due to the major grade of lipophilicity [41] and effectively cross the blood-brain barrier [13]. Considerations regarding the bioavailability and tissue concentration are not restricted to cancer cells but involve exposure of tumour surrounding tissue especially in the case of topically administered therapy. The effect of SMO antagonists in tumour stroma is largely unknown. Frequently, tumour-associated stromal cells not only provide paracrine factors but also present a coordinated regulation of major oncogenic intracellular signalling [42,43,44]. A large number of tumours have been demonstrated associated with Hh peptide secretion by tumour cells themselves and by the stromal microenvironment [42,45,46]. Moreover, two different studies demonstrated that CAFs can promote epithelial-to-mesenchymal transition through the activation of the Hh pathway [47,48]. On the other hand, NBCCS fibroblasts carrying chronic active intracellular Hh signal transduction, functionally exhibit CAF features [49,50] underling the concept that Hh signalling deregulation may represent the joint point of similarity between CAFs and NBCCS fibroblasts.

This project, based on the idea that dermal-epidermal interaction plays a crucial role in BCC onset and progression, explored the possibility that inhibition of Hh signalling could be effective in targeting tumour-prone fibroblasts in the context of subjects predisposed to skin cancer and BCC-CAFs.

## 2. Materials and Methods

### 2.1. Isolation of Primary Fibroblastic Population from Human Tissue Skin

NBCCS patients were diagnosed by clinical examination and family history and then genetically characterized by genomic DNA sequencing. Tissue samples were collected from NBCCS (unaffected skin at the time of surgical operation, n = 10; age range 32–78, mean age 57.7 ± 16 years), BCCs (tumoral tissue, n = 7; age range 66–87, mean age 74.4 ± 6.9 years) and control healthy donors (n = 12, age range 29–96, mean age 61.2 ± 22.6 years). Skin fragments obtained from surgery, were catted into approximately 4 mm^2^ sized pieces and digested overnight at 4 °C with dispase (2.5 mg/mL) to separate epidermis from dermis. Dermis was digested with collagenase for 2 h at 37 °C and extracted normal human fibroblasts (NHFs), CAFs and NBCCS-human fibroblasts (NBCCS-HFs) were maintained in culture with DMEM (EuroClone S.p.A., Milan, Italy) supplemented with 10% FBS and antibiotics (Hyclone Laboratories, South Logan, UT, USA). NBCCS patient characteristics and corresponding *PTCH1* mutation are reported in Table 1. BCC patient and healthy donor characteristics are reported in Supplementary Tables S1 and S2. Vismodegib (GDC0449) and sonidegib (LDE225) were both purchased from MedChemExpress (MedCheExpress LLC, NJ, USA) and Adooq Biosciences (Irvine, CA, USA) for comparison.

### 2.2. Nevoid Basal Cell Carcinoma Syndrome (NBCCS) Patients’ Genomic Characterization

*PTCH1* (NM_000264.3) mutations were identified in the genome from non-tumoral tissue (fibroblasts or blood cells). Genomic DNA was extracted using Tissue Kit (Qiagen, Milan, Italy) following the manufacturer’s instructions. About 100–200 ng of genomic DNA was subject to PCR in a total volume of 50 μL containing 25 μL of 2× AmpliTaq Gold^TM^ 360 Master Mix (Thermo Fisher Scientific, Monza, Italy) and 22 sets of primers (25 pmol each). Primer sets were designed to cover the entire coding sequences plus a few nucleotides into the intron sequences on both ends. PCR primer sequences are available upon request. DNA fragments were checked by electrophoresis in 2% agarose gel and purified before bidirectional direct Sanger sequencing and chromatograms inspection using ChromasPro software. Variants that changed their nucleotide sequence, to assess the pathogenicity data, were matched to https://varsome.com, https://clinvarminer.genetics.utah.edu and https://www.lovd.nl databases (accessed on 20 June 2021).

### 2.3. MMT Assays

Briefly, 0.8 × 10^4^ fibroblasts were seeded into the 24-well plates for 24 h to adhere. Then, growth medium was changed with fresh medium containing treatments (or not for control cells) at the appropriate concentrations. At the experimental end point (72 h), cells were incubated with 3-(4,5 dimethylthiazol)-2,5-diphenyl tetrazolium bromide (MTT) for 2 h. After this time, the medium was removed and the resulting crystals were solubilized in DMSO. The absorbance was measured at 570 nm with a reference wavelength of 690 nm. Absorbance readings were subtracted from the value of blank wells, and results were calculated as a percentage of absorbance with respect to control samples. Experiments were performed in duplicate.

### 2.4. Flow Cytometry Analysis

Cell death and apoptosis were analysed by the annexin-V FITC/propidium iodide (PI) double staining method after 48 h of treatment. Cells were harvested by trypsinization, suspended in the staining buffer (10 mm HEPES ⁄ NaOH, pH 7.4, 140 mm NaCl, 2.5 mm CaCl_2_), stained with FITC-labeled annexin V and PI for 15 min at RT in the dark and then kept on ice until analysis; 20,000/sample cells were analysed using a MACSQuant 10 instrument. Data were interpreted with MACSQuantify software (Milthenyi Biotech, S.r.l., Bologna, Italy).

### 2.5. Semi-Quantitative Real-Time Polymerase Chain Reaction (RT-PCR) and Gene Expression Array Cards Analysis

Total RNA was extracted using Aurum Total mini kit (BioRad, Milan Italy). cDNA was synthesized from 1 μg of total RNA using the FirstAid kit (Fermentas, ThermoFisher Scientific, Waltham, MA, USA) and loaded on 384-well microfluidic cards designed to perform probe-based TaqMan real-time PCR on an Applied Biosystems^®^ QuantStudio^TM^ 7 Flex instrument (Thermo Fisher Scientific, Monza, Italy). Cards were configured with selected primers and probe sets to analyze 90 target genes and three housekeeping genes (18s rrna, gliceraldeide-3-fosfato deidrogenasi (*GAPDH*) and β-actin). Results were evaluated using cloud-based platform software (ThermoFisher Scientific). For semi-quantitative real-time PCR, cDNA were amplified using SsoAdvanced Universal Syber Green Supermix (BioRad) containing 25 pmol of forward and reverse primers using a CXF96 Touch Cycler (BioRad). All samples were tested in triplicate. Amplification of the β-actin transcript from each sample was included as internal control. Sequences of primers (intron spanning) are given separately in the Supporting Information (Table S3). 

### 2.6. Western Blot Analysis

Prepared cells were lysed with RIPA buffer containing proteases and phosphatases inhibitors. Proteins were separated on SDS-polyacrylamide gels, transferred to nitrocellulose membranes and then treated with the following primary antibodies: mouse monoclonal anti-β-catenin (Santa Cruz Biotechnology Inc.), rabbit polyclonal pSer473-AKT and pSer9-GSK3β (Cell Signalling Technology, MA, USA). Anti-β-actin mouse monoclonal antibody (Sigma Aldrich, Merck KGaA, Darmstadt, Germany) was used to normalize protein content. Horseradish peroxide-conjugated goat anti-mouse and goat anti-rabbit antibodies complexes were detected by chemiluminescence (Cell Signalling Technology). Imaging and densitometric analysis were performed with a UVITEC Mini HD9 acquisition system (Alliance UVItec Ltd., Cambridge, UK).

### 2.7. Immunofluorescence Analysis

Cells on coverslips were fixed with 4% paraformaldehyde for 15 min at room temperature followed by 0.1% Triton X-100 to allow cell permeabilization. Cells were then incubated with anti-α-SMA monoclonal (Sigma Aldrich, Merck Life Science S.r.l. Milan, Italy), or anti-FAP rabbit (Cohesion Bioscience, London, UK) for 1 h. Primary antibodies were visualized using an anti-mouse or anti-rabbit IgG Alexa Fluor 488 (BD Biosciences, Milan, Italy). Fluorescence signals were recorded using a CCD camera (Zeiss, Oberkochen, Germany).

### 2.8. Cytokines Protein Array

The expression of 20 human cytokines and 41 growth factors were analysed using a commercially available antibody array system (RayBio^®^ C-Series Human Inflammation Array C1 Map, and C-Series Human Growth Factors Array C1 RayBiotech, Inc. Peachtree Corners, GA, USA) that uses membrane-bound cytokine-specific antibodies to capture proteins in biological fluids. The procedure was performed according to the manufacturer’s instructions. Cells were seeded in 10 cm culture dishes and treated (or not) with vismodegib and sonidegib 10 µM for 72 h. After removing the drug, cells (and control untreated proliferating fibroblasts) were maintained in serum-free medium for 48 h before collect conditioned medium. The cytokine array membranes were blocked in 1 × blocking buffer for 30 min at room temperature (RT) and then were incubated with 1 mL of conditioned medium at 4 °C overnight. The medium was then decanted from each container, and the membranes were washed three times with 1 × wash buffer I, followed by two washes with 1 × wash buffer II at RT. Next, the membranes were incubated in biotin-conjugated primary antibodies for 2 h at RT and then washed as described above before incubation in 1:1000-diluted horseradish peroxidase-conjugated streptavidin for 2 h. The membranes were then washed thoroughly and incubated with a chemiluminescent ECL substrate at RT for 5 min. Imaging and densitometric analysis were performed with a UVITEC Mini HD9 acquisition system (Alliance UVItec Ldt, Cambridge, UK).

### 2.9. Quantification of Vismodegib and Sonidegib Uptake

Cell uptake was determined in three independent experiments performed in three different cell lines. Cells were harvested and counted after 72 h treatments and stored at –80°C until analysis. Cell pellets were extracted after three freezing/thawing cycles in liquid nitrogen and addition of 200 pmoles of d31Cer[NS] 34:0 as the internal standard (iSTD) added in isopropanol containing 0.001% BHT. The cell suspension was extracted twice with 1 mL of ethyl acetate. The upper organic phase was evaporated under nitrogen and the dry extract was dissolved in 200 µL isopropyl alcohol before injection. Calibration curves were prepared with the vismodegib and sonidegib authentic compounds in the concentration range 2–400 nM with the iSTD used for the extraction. Vismodegib and sonidegb were quantified with reversed phase liquid chromatography coupled to a time-of-flight mass spectrometry (LCMS) system (Agilent Technologies, Santa Clara, CA, USA). For the quantitation, the protonated ions [M + H]+ were extracted for vismodegib (m/z 421,0175), sonidegib (m/z 486,1999), and the iSTD (m/z 568,7077). The method was linear (R > 0.9999) in the concentration range between 2–400 nM for both vismodegib and sonidegib.

### 2.10. Statistical Analysis

Results in the figures are representative of several experiments we performed with at least five cell lines from different donors for each cell type. Quantitative data were reported as mean ± standard deviation (SD). Student’s *t*-test was used to assess statistical significance with thresholds of * *p* ≤ 0.05 and ** *p* ≤ 0.01. Array card results reported in Table 2, Table 3 and Table 4 were obtained with a one-way ANOVA statistical test with thresholds >2.0 and <0.5 fold-change and *p* value < 0.05.

### 2.11. Ethics Statement

This study was approved by the Institutional Research Ethics Committee (Istituti Regina Elena e San Gallicano), Rome, Italy, and was performed after informed written consent to collect samples of human material for research.

## 3. Results

### 3.1. Patients’ Demographic and Genetic Characterization

A total of 10 NBCCS patients were included in the study. Patients were first diagnosed by clinical examination and family history, and then the genetic characterization confirmed Gorlin Syndrome diagnosis. Consistent with previous evidences demonstrating the absence of hot spot mutation in *PTCH1* gene [51,52,53,54], molecular analysis of patients enclosed in this study recorded heterozygotic genetic variation distributed along the entire coding sequence (Table 1). Among these mutations, eight were missense changes resulting in harmful or pathogenic effects based on data from online databases (https://varsome.com, https://clinvarminer.genetics.utah.edu and https://www.lovd.nl, accessed on 20 June 2021), 1 was a deletion resulted in a frameshift of the coding sequence, and one insertion of a surplus CGG triplet was at the site of seven times CGG repeat in a functionally important 5′UTR intron region involved in *PTCH1b* variant transcription and translation [55,56].

**Table 1:** Mutations found in nevoid basal cell carcinoma syndrome (NBCCS) cases examined.

Patients	Age	Sex	Affected Exon	DNA/Protein Change	Effect on Sequence
NBCCS-HF1	63	F	Exon 17	c.2635G > T p.Asp879Tyr	missense
NBCCS-HF2	76	M	Exon 12	c.1510T > C (p.Leu503Ser)#	missense
NBCCS-HF6	25	F	Exon 10	c.1309G > A (p.Val437Ile)	missense
NBCCS-HF7	57	F	Exon 15	c.1510T> C (p.Leu503Ser)#	missense
NBCCS-HF8	64	F	Exon 5	c.653delA (p.Gln218Gln *fs*X219)	deletion
NBCCS-HF9	69	M	Exon 1	c.113G > T (p.Gly38Val)	missense
NBCCS-HF10	78	F	Exon 24	c.4172G > A (p.Arg1391Gln)	missense
NBCCS-HF11	32	M	Exon 10	c.1309G > A p.Val437Ile	missense
NBCCS-HF12	57	F	Exon 2	c.-6_-4dupGGC[1]	5′ untranslated region cis-regulatory element
NBCCS-HF13	56	M	Exon 19	c.3227T > C (p.Ile1076Ser)	missense

Mutation analysis of *PTCH1* gene was performed by direct sequencing. Data were matched to https://varsome.com, https://clinvarminer.genetics.utah.edu and https://www.lovd.nl (accessed on 20 June 2021) databases to assess the pathogenicity of gene variants. # Indicated patients of the same family.

### 3.2. Comparative In Vitro Evaluation of Vismodegib and Sonidegib on Fibroblasts Proliferation

**Figure 1:**
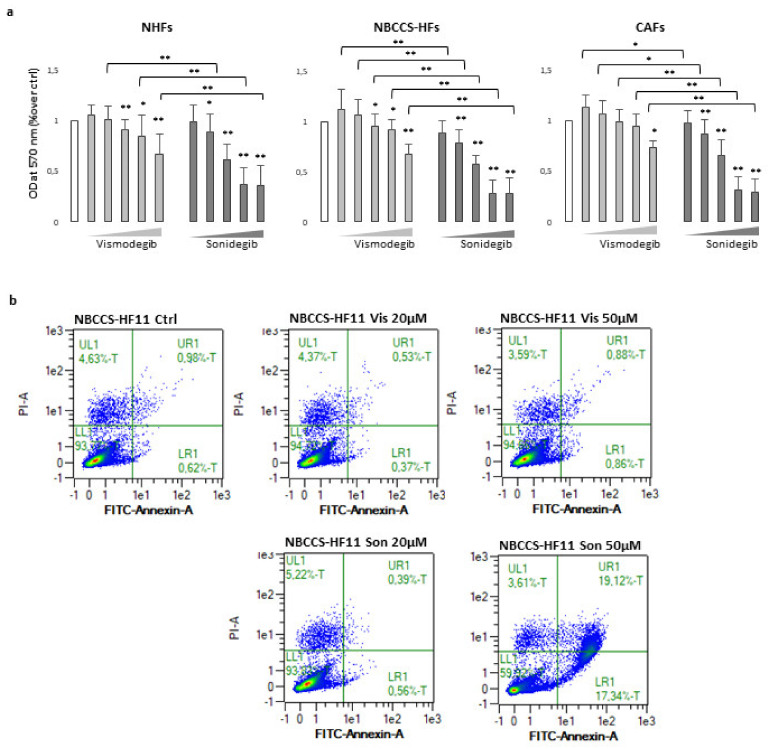
(**a**) MTT assay of NHFs (n = 11), NBCCS-HFs (n = 9) and CAFs (n = 6) after 72 h treatment with vismodegib and sonidegib (range 5–100 μM). Data are reported as mean ± SD. Experiments were performed in duplicate. (**b**) AnnexinV/iodide propidium staining evidenced apoptosis cell death only with sonidegib treatment. Dot plots show one representative experiment performed 48 h after Hh inhibitors treatment. * *p* ≤ 0.05 and ** *p* ≤ 0.01.

To evaluate the cytotoxicity of SMO antagonist, a 72 h MTT assay was performed in NHFs, NBCCS-HFs and CAFs. No obvious cytotoxicity was observed with vismodegib at all the concentration tested. However, with higher concentrations used (20–100 µM), it reduced cell proliferation of every cell types analysed in a dose-dependent manner. By contrast, sonidegib exerted a global stronger effect leading to a progressive decrease of the proliferation rate with lower doses and near to complete dead of cell culture exposed to 50 µM and 100 µM (Figure 1a). Differences in vismodegib and sonidegib toxicity were confirmed by quantification of apoptotic rate by Annexin V/PI staining (Figure 1b). Overall, no significant differences emerged comparing sensibility to both Hh inhibitors in normal, syndromic and BCC-associated fibroblasts. Furthermore, the specificity of SMO-dependent effect was confirmed by small interfering RNA (si-RNA) experiments. In this case, a mean 0.68 ± 0.22 and 0.53 ± 0.16 reduction in SMO mRNA in NHFs and CAFs respectively, impacted on cell proliferation compared to non-specific (NS-RNA). By contrast, syndromic fibroblasts with comparable decrease level of SMO mRNA (0.45 ± 0.02), showed no change in the MTT assay (Supplementary Figure S1a).

### 3.3. Analysis of Smoothened (SMO) Antagonists on Normal Human Fibroblasts (NHFs), NBCCS-HFs and CAFs Gene Expression Profile

**Figure 2:**
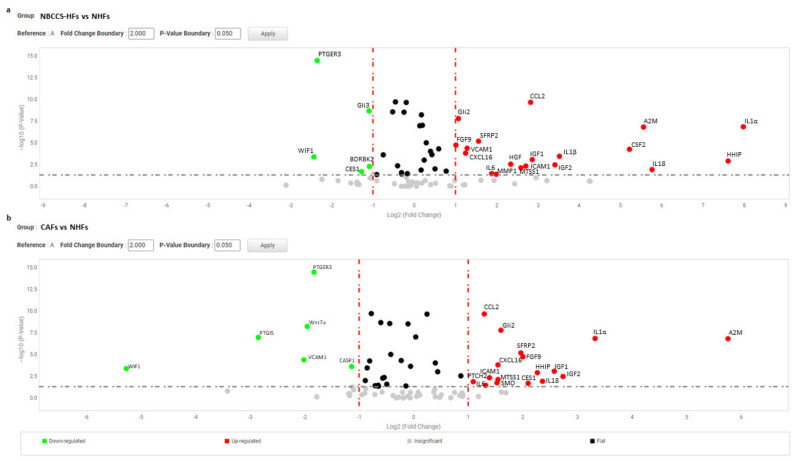
(**a**) Volcano plot represents basal gene expression analysis comparing NBCCS-HFs (n = 9) mRNA to NHFs (n = 9) mRNA level of expression. (**b**) Volcano plot represents basal gene expression analysis comparing CAFs (n = 6) mRNA to NHFs (n = 9) mRNA level of expression. Analysis was performed combining three different housekeeping genes (β-actin, GAPDH and 18 s). One-way ANOVA statistical test with thresholds >2.0 fold-change and *p* < 0.05 defined significant increase are reported in red; significant decrease <0.5 fold-change and *p* < 0.05 reported in green; any fold-difference with *p* ≥ 0.5, e.g., insignificant are reported in grey; ≤2.0 or ≥0.5 difference e.g., flat reported in black.

To further evaluate the biological effect of Hh inhibitors on fibroblasts avowing acute cell damage, was selected the dose of 10 µM. We compared the gene expression profile of 9 independent NHFs strains and 9 NBCCS-HFs treated (or not) with vismodegib and sonidegib for 72 h. The study also included 6 BCC-associated CAF cell lines under the same treatment regime. A total of 93 genes of interest related to the Hh pathway, Wnt pathway and to pro-tumoral fibroblast phenotype were evaluated using the gene expression array card system. First, to select genes dissimilarly expressed in untreated cells at basal level, samples were clustered in three different biogroups according to the donor type (NHF, NBCCS-HF and CAF). The level of mRNAs in healthy fibroblasts was used as reference and arbitrarily indicated as 1.0. Among selected genes were identified 24 mRNAs with over two-fold significant difference (>2.0 and *p*-value < 0.05) in expression levels between NBCCS and NHF samples (19 up-regulated and 5 down-regulated) (Table 2 and Figure 2).

**Table 2:** Gene expression analysis, 72 h.

Gene	NBCCS-HF	NBCCS-HF	NBCCS-HF	CAF	CAF	CAF	NHF	NHF	NHF
		Vis	Son		Vis	Son		Vis	Son
*IL1α*	252.3	48.1	23.5	10.0	2.1	6.1	1.0	1.0	1.7
*HHIP*	195.5	122.9	42.7	4.8	6.0	1.9	1.0	0.32	0.54
*IL18*	54.4	10.9	11.5	5.1	13.5	20.0	1.0	2.7	2.7
*A2M*	47.1	21.2	8.4	54.2	57.9	52.8	1.0	1.4	2.2
*CSF2*	13.6	6.7	28.4	0.57	0.29	0.62	1.0	1.3	0.52
*IGF2*	10.6	6.6	11.8	7.4	7.6	8.4	1.0	0.87	0.96
*CCL5*	9.3	4.3	3.6	3.4	3.8	3.4	1.0	1.3	1.6
*IGF1*	7.3	4.7	8.0	6.6	6.7	6.8	1.0	0.63	1.3
*CCL2*	7.1	7.2	6.9	2.5	1.7	1.8	1.0	0.58	0.59
*ICAM1*	6.5	4.4	4.1	2.6	3.2	2.7	1.0	0.95	0.7
*MTSS1*	6.0	5.5	3.9	2.9	2.3	2.8	1.0	0.83	0.32
*HGF*	5.1	2.5	2.5	2.0	0.87	1.6	1.0	0.82	0.71
*MMP1*	4.0	2.7	3.3	0.91	0.53	1.1	1.0	0.96	0.61
*IL6*	3.7	3.6	3.3	2.5	2.3	2.4	1.0	1.1	0.94
*IL1β*	11.5	3.9	3.7	0.55	0.19	0.60	1.0	0.32	0.17
*SFRP2*	2.9	7.5	9.1	3.9	5.4	4.1	1.0	0.42	0.32
*VCAM1*	2.4	10.1	6.9	0.25	2.1	1.1	1.0	0.59	0.29
*CXCL16*	2.4	4.5	3.7	2.9	2.8	2.8	1.0	0.74	0.98
*CSF3*	1.2	6.1	5.5	0.75	0.27	0.12	1.0	2.1	0.34
*Gli2*	2.1	0.7	1.9	3.0	3.3	1.9	1.0	1.5	1.4
*FGF9*	2.0	2.2	3.0	4.0	2.2	2.0	1.0	1.0	1.5
*PTCH2*	1.1	1.5	1.4	2.1	1.8	2.4	1.0	1.8	4.9
*WIF1*	0.18	0.46	0.75	0.03	0.03	0.02	1.0	2.1	0.08
*PTGER3*	0.2	0.57	0.45	0.28	0.24	0.22	1.0	0.82	0.7
*CES1*	0.41	1.6	2.0	4.3	3.4	3.5	1.0	1.3	0.8
*Gli3*	0.47	0.64	0.84	0.66	0.58	0.64	1.0	1.0	0.91
*BDKRB2*	0.47	0.54	0.63	0.67	0.56	0.57	1.0	0.72	0.62
*SMO*	1.7	0.77	0.87	2.9	1.7	1.4	1.0	0.79	1.4
*Wnt7a*	1.1	1.6	3.25	0.26	0.28	0.31	1.0	0.54	0.57
*CASP1*	0.59	0.9	0.69	0.45	0.38	0.35	1.0	1.0	0.65
*PTGIS*	1.1	1.7	1.1	0.14	0.16	0.19	1.0	0.68	0.97
*CD40*	1.3	1.6	2.7	0.96	0.75	1.0	1.0	0.77	0.59
*LTC4S*	0.87	0.62	2.3	1.3	1.1	1.3	1.0	0.85	1.6

Gene expression analysis NBCCS-HFs and CAFs compared to NHFs. Data also report the effect of 72 h treatment with vismodegib and sonidegib (10 µM). Significantly overexpressed mRNA > 2.0 fold-change and *p* < 0.5 are marked in red, whereas significantly downregulated mRNA < 0.5 fold-change and *p* < 0.5 are marked in blue. The level of mRNAs in healthy fibroblasts was used as reference and arbitrarily indicated as 1.0.

**Figure 3:**
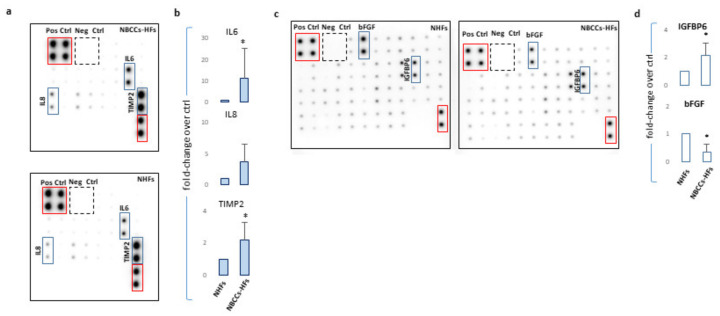
(**a**) One representative protein array assay comparing inflammatory factors released by NBCCS-HFs and NHFs. (**b**) Histograms report densitometric analysis of four independent experiments mean ± SD. (**c**) One representative protein array assay comparing growth factors released by NBCCS-HFs and NHFs. (**d**) Histograms report densitometric analysis of three independent experiments mean ± SD. * *p* ≤ 0.05.

Six transcripts correspond to genes involved in inflammation (IL1α, IL18, IL1β, IL6, CXCL16, and CCL2), and four mRNAs correspond to growth factors (FGF9, IGF1, IGF2 and HGF). In addition, members of the immunoglobulin superfamily of endothelial adhesion molecules, vascular cell adhesion molecule (VCAM-1), intercellular cell adhesion molecule (ICAM-1), and colony-stimulating factors 2 (CSF2) which stimulate granulocytes and macrophages were increased. Interestingly, Alpha-2-Macroglobulin (A2M) a protease inhibitor and cytokine transporter resulted significantly higher. Due to the capacity of A2M to disrupt inflammatory cascade, its high expression could be explained by the marked pro-inflammatory profile of Gorlin’s patient fibroblasts. Human hedgehog interacting protein (HHIP), a negative regulator of Hh signalling frequently underexpressed in several tumours [57,58,59,60], showed an extremely high level of expression in NBCCS-HFs suggesting a compensatory mechanism acting on Hh pathway. Moreover, our data confirmed previous studies [49,50,51] demonstrating high level of matrix metalloproteinase 1 (MMP1) and of Secreted Frizzled Related Protein 2 (SFRP2), a soluble modulator of Wnt signalling. By contrast, Wnt Inhibitory Factor 1 (WIF1) an inhibitor of the same pathway, Prostaglandin E Receptor 3 (PTGER3), and Bradykinin receptor B2 (BDKRB2) were significantly lower (fold-decrease < 0.5 and *p* < 0.05) in NBCCS-HFs than in normal fibroblasts (Table 2). Another sonic hedgehog target gene, metastasis suppressor 1 (MTSS1), a cytoskeletal associated protein implicated in cell motility and invasiveness [61,62,63], resulted significantly up-modulated in syndromic fibroblasts. Interestingly, MTSS1 is involved in carcinogenesis not only due to an augmented metastatic capability but also because MTSS1 serves as a co-transcription factor by binding to GLI proteins enhancing Hh target genes transcription [64]. Carboxylesterase1 (CES1), an enzyme involved in lipids hydrolysis, lipoproteins assembly and fatty acyl and cholesterol ester metabolism [54], also implicated in tumour-killing activity of monocytes [65] showed significant lower level in syndromic fibroblasts. A pronounced pro-inflammatory signature of Gorlin syndrome cells was additionally evidenced by the higher mRNA level of several other interleukins and cytokines (CXCL10, IL8, CCL5, CCL19, TNFα and PTGS2) even if this results set did not fully reach statistical significance (>2.0 and *p* ≥ 0.05 e.g., insignificant). GLI2 and GLI3 displayed opposite regulation (up- and down-regulation, respectively). This is in line with the idea that GLI2 is a positive regulator (activated by SHH) whereas GLI3 is a repressor (reduced by Shh) of Hh targets [6,66]. Overall mRNA profile proven stable Hh over-activation in fibroblasts carrying heterozygotic *PTCH1* mutation that persists in vitro. The protein array system used to compare some of the inflammatory proteins released in culture medium by healthy and syndromic cells confirmed differences in IL6 expression (fold-increase 11.3 ± 13.9; *p* = 0.032) and CXCL8 (fold-increase 3.72 ± 2.8; *p* = 0.055) (Figure 3a,b) and closely correlated to the mRNA results (Table 2 for IL6 and Table 3 for CXCL8).

Nevertheless, in the case of IL1α and β the corresponding proteins were barely revealed with this detection method. Secretion of TIMP2 protein, a modulator of MMPs, was also significantly higher (2.19 ± 1.1 *p* = 0.046) in syndromic cells confirming the propensity of NBCCS-HFs to actively remodel the extracellular matrix. Similarly, an array panel for the detection of some mitogenic peptides revealed lessened basic fibroblast growth factor (bFGF) (fold-decrease 0.33 ± 0.30 *p* = 0.014) and higher insulin growth factor binding protein 6 (IGFBP6; fold-increase 2.1 ± 0.9 *p* = 0.049) in NBCCS-HFs with respect to normal fibroblasts (Figure 3c,d). Interestingly, CAFs isolated from sporadic BCCs showed an overall expression profile similar to NBCCS-HFs (Table 2). Among the 19 mRNAs up-regulated in syndromic fibroblasts, 15 demonstrated analogous modulation in BCC-associated fibroblasts (IL1α, CCL2, CXCL16, IL6, IL18, ICAM, A2M, SFRP2, FGF9, IGF2, IGF1, HGF, HHIP, MTSS1 and GLI2). As is likely in syndromic patients, CAFs show a moderate increase of additive inflammatory markers (CCL5, and CXCL10 e.g., insignificant). Significant up-modulation of PTCH2 and SMO transcripts confirmed the activation of Hh signalling in BCC-associated CAFs and suggested this anomaly as a possible priming mechanism for Hh signalling activation in absence of genetic aberration. In line with this idea, Walter and collaborators reported SMO overexpression as a mechanism for the activation of Hh signalling in human pancreatic CAFs [48]. NBCCS-HFs and CAFs also share the reduced expression of WIF1 and PTGER3. CAFs additionally presented significant lower level of Wnt7a, caspase-1 and prostacyclin synthase (PTGIS) compared to normal fibroblasts. By contrast with syndromic fibroblasts, CAFs express augmented level of CES1. More data regarding level of mRNA expression uniformly expressed in normal and pathological cells are summarized in Table 3.

**Table 3:** Gene expression analysis, unmodified genes.

Gene	NBCCS-HF	NBCCS-HF	NBCCS-HF	CAF	CAF	CAF	NHF	NHF	NHF
		Vis	Son		Vis	Son		Vis	Son
*a-SMA*	0.73	0.88	1.0	0.58	0.54	0.63	1.0	1.0	0.86
*FAP*	1.1	1.23	1.8	1.0	0.86	0.94	1.0	1.2	0.98
*Wnt1*	0.98	3.1	0.98	0.44	0.47	0.30	1.0	0.84	0.54
*Wnt2b*	1.2	0.96	0.77	0.87	0.82	0.92	1.0	1.1	1.1
*Wnt5a*	1.5	1.2	1.5	0.2	0.42	0.47	1.0	1.0	1.0
*Wnt6*	3.1	1.2	12.3	0.67	0.34	0.95	1.0	0.59	0.76
*Wnt9a*	0.9	0.40	0.25	0.88	0.88	0.88	1.0	0.51	0.75
*Wnt9b*	0.01	0.22	0.27	1.0	0.22	0.12	1.0	0.17	0.53
*Wnt10b*	0.21	0.54	0.21	0.09	0.51	0.122	1.0	0.85	0.59
*Wnt11*	0.53	1.6	1.6	0.64	0.61	0.31	1.0	0.74	0.8
*Wnt16*	0.38	1.2	3.5	0.44	0.48	0.53	1.0	1.4	1.3
*TIMP1*	1.8	1.4	1.9	1.4	0.89	1.7	1.0	1.3	1.2
*TIMP2*	0.84	1.1	1.3	0.93	1.0	1.0	1.0	1.1	1.0
*TGFβ1*	0.72	1.1	0.77	0.77	0.77	1.2	1.0	1.0	0.97
*VEGFA*	1.3	1.4	1.9	1.3	1.2	1.2	1.0	0.83	1.0
*TNFSF13B*	1.85	2.76	1.86	1.6	1.9	1.5	1.0	0.98	1.2
*TNFSF1B*	0.40	0.80	0.64	0.93	1.3	1.7	1.0	0.43	0.37
*TNFSF1A*	0.91	0.78	1.2	0.54	0.49	0.49	1.0	0.63	0.75
*TNF*	3.8	6.1	11.9	1.1	1.4	1.7	1.0	0.88	0.52
*SUFU*	1.0	1.0	1.0	0.96	0.99	0.94	1.0	1.1	1.1
*SMAD7*	0.94	1.8	1.9	1.1	0.97	1.1	1.0	0.13	0.58
*SMAD3*	1.9	1.3	1.5	1.3	1.3	1.3	1.0	1.1	1.2
*SHH*	0.11	0.75	1.1	0.29	1.1	0.16	1.0	0.61	4.2
*SFRP1*	1.1	0.34	0.47	1.9	1.2	1.9	1.0	0.77	0.25
*PTGS2*	5.4	5.1	2.6	1.8	1.9	1.6	1.0	0.83	0.43
*PTGS1*	1.4	1.0	1.3	0.54	0.51	0.36	1.0	0.91	0.76
*PTGIR*	1.5	1.2	2.1	1.04	1.1	1.2	1.0	2.1	1.2
*PTGFR*	0.38	0.59	0.89	0.7	0.77	0.59	1.0	1.1	1.2
*PTGER2*	0.86	1.3	1.7	0.46	0.54	0.32	1.0	0.41	0.30
*PTCH1*	0.92	0.69	0.92	1.3	1.4	1.3	1.0	1.0	0.95
*PLA2G1B*	0.28	0.30	0.38	0.96	0.06	1.5	1.0	0.46	0.30
*PDE4B*	0.48	0.59	0.67	0.50	0.44	0.49	1.0	0.93	0.97
*PDE4A*	0.7	1.2	1.8	1.0	1.1	1.1	1.0	1.0	0.86
*MMP3*	0.86	1.0	1.8	0.74	0.6	0.54	1.0	1.2	0.81
*MAPK8*	0.81	0.80	1.0	0.80	0.81	0.86	1.0	1.0	0.85
*MAPK3*	0.86	0.88	1.1	0.87	0.89	1.1	1.0	0.98	1.1
*MAPK14*	0.76	0.89	1.1	0.69	0.67	0.72	1.0	0.95	0.99
*MAPK1*	0.89	0.91	0.92	0.64	0.65	0.62	1.0	0.81	0.74
*IL7*	0.34	0.76	3.4	0.52	0.96	1.5	1.0	0.95	1.2
*IGFBP6*	1.0	1.3	1.9	0.61	0.67	0.90	1.0	1.0	1.6
*IGFBP3*	0.89	0.98	1.6	0.64	0.50	0.69	1.0	0.80	1.2
*Gli1*	1.4	1.2	0.32	1.4	1.9	0.94	1.0	1.7	1.2
*FGF7*	0.98	0.93	1.5	1.1	2.4	1.4	1.0	1.2	1.1
*FGF2*	1.2	1.2	1.1	1.4	1.2	1.6	1.0	0.86	1.2
*FAP*	1.1	1.2	1.8	1.03	0.86	0.94	1.0	1.2	0.98
*EGF*	2.3	0.65	0.59	2.3	1.7	0.32	1.0	2.7	0.30
*DKK3*	0.87	0.59	0.71	0.54	0.53	0.60	1.0	0.96	1.2
*DKK1*	0.53	0.72	0.91	1.7	0.77	0.59	1.0	0.8	0.93
*CXCL8*	19.2	7.4	9.5	1.8	1.4	0.82	1.0	0.7	1.4
*CXCL10*	3.2	3.9	14.1	19.0	3.3	1.3	1.0	2.8	1.4
*CTNNB1*	0.94	1.5	1.8	0.82	1.0	1.1	1.0	1.2	1.0
*CSF1*	0.71	0.97	1.1	0.81	0.72	0.79	1.0	1.1	1.0
*COL1A2*	0.58	0.99	1.1	1.2	0.66	0.62	1.0	1.3	1.6
*COL1A1*	1.2	0.88	0.93	1.1	1.6	1.4	1.0	1.3	1.4
*CCNB1*	1.5	1.8	1.2	0.85	0.89	0.53	1.0	0.86	0.65
*CCL3*	0.38	1.2	3.5	0.44	0.48	0.91	1.0	1.4	1.4
*CCL19*	7.1	0.64	6.1	1.3	0.24	0.64	1.0	1.7	1.8
*CACNA2D1*	0.86	1.3	1.2	2.2	1.2	1.6	1.0	1.5	1.0
*CACNA1C*	0.95	1.1	0.64	1.3	1.3	1.4	1.0	1.2	1.2
*BDKRB1*	0.48	0.92	1.1	0.47	0.48	0.37	1.0	1.3	0.77

Summary of data regarding mRNA that resulted similarly (e.g., insignificant or flat) expressed in NHFs, NBCCS-HFs and CAFs at basal level or that are not significantly modified by SMO inhibitors treatment (72 h). The level of mRNAs in healthy fibroblasts was used as reference and arbitrarily indicated as 1.0.

**Figure 4:**
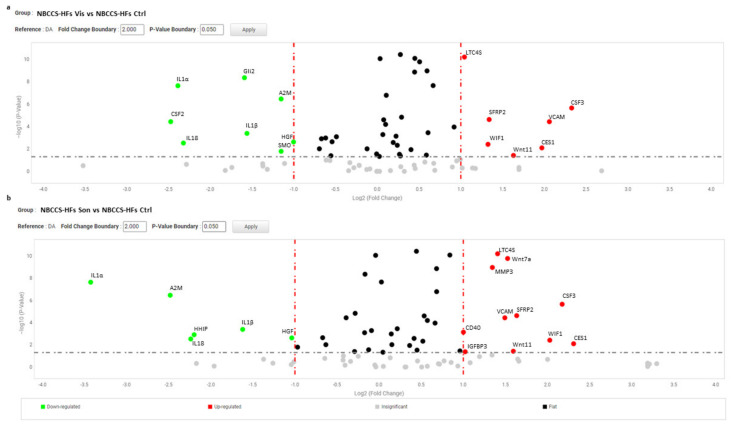
Volcano plot represents gene expression analysis comparing NBCCS-HFs treated with vismodegib (**a**) and sonidegib (**b**) for 72 h (n = 9) to NBCCS-HFs untreated (n = 9). (b) Analysis was performed using three different housekeeping genes (β-actin, GAPDH and 18S). A one-way ANOVA statistical test with thresholds >2.0 fold-change and *p* < 0.05 defined significant increase are reported in red; significant decrease <0.5 fold-change and *p* < 0.05 reported in green; any fold-difference with *p* ≥ 0.5, e.g., insignificant are reported in grey; ≤2.0 or ≥0.5 difference e.g., flat reported in black.

For further statistical analysis, data were pooled in 9 separated biogroups according to cell cultures origin and treatment regime. After 72 h, inhibition of SMO partially reverted the above described “Gorlin syndrome fibroblast expression profile” (Table 2). Nonetheless, most of the deregulated genes did not reached expression level similar to healthy fibroblasts. Notably, only the mRNA level of GLI2 was fully normalized by SMO antagonists. In detail, both vismodegib and sonidegib significantly (two-fold difference and *p* < 0.05 NBCCS-HFs-treated vs. untreated) counteract deregulation of IL1α, IL1β, IL18, HGF, A2M (by decreasing), CES1 and WIF1 (by increasing) in NBCCS-HFs (Table 2 and Figure 4) that, however, resulted still significantly higher compared to healthy fibroblasts.

**Figure 5:**
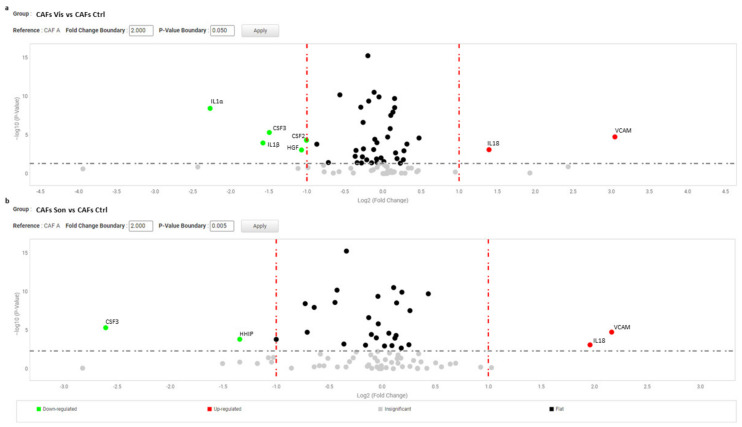
Volcano plot represents gene expression analysis comparing CAFs treated with vismodegib (**a**) and sonidegib (**b**) for 72 h (n = 6) to CAFs untreated (n = 6). (b) Analysis was performed using three different housekeeping genes (β-actin, GAPDH and 18S). One-way ANOVA statistical test with thresholds >2.0 fold-change and *p* < 0.05 defined significant increase are reported in red; significant decrease <0.5 fold-change and *p* < 0.05 reported in green; any fold-difference with *p* ≥ 0.5, e.g., insignificant are reported in grey; ≤2.0 or ≥0.5 difference e.g., flat reported in black.

Additionally, hyper expression of HHIP, ICAM, MTSS1, MMP1 resulted attenuated by treatments (Table 2). By contrast, the presence of Hh inhibitors exacerbated SFRP2 and VCAM1 overexpression. Also transcripts of *Wnt11*, *CSF3* and leukotriene synthase 4 (*LTC4S*), comparable at basal level in NBCCS-HFs and healthy fibroblasts, was stimulated by both compounds. Once again, in line with MTT assay results, sonidegib exerted a comprehensive stronger effect compared to vismodegib. For example, focusing on mRNA variation of the four highest up-modulated genes and responsive to the activity of both molecules (IL1α, HHIP, IL1β, and A2M), we observed a mean fold-change of 0.37 ± 0.18 and 0.17 ± 0.05 with vismodegib and sonidegib respectively. Reversion of WIF1, PTGER3, CES1, GLI3 and BDKRB2 hypo-expression in the presence of vismodegib or sonidegib was extremely similar with both drugs (2.36 ± 1.0 and 2.88 ± 1.39). Surprisingly, in CAFs pharmacological inhibition of Hh pathway exerted a moderate effect on the pro-tumorigenic profile (Table 2). IGF1, ICAM, MTSS1, IL6, SFRP2, and CXCL16, were unaffected by treatments and still resulted significantly higher than in normal fibroblasts. Under treatment with both SMO antagonists, IL1α, CCL2, HGF, FGF9 and ICAM1 mRNA tended to expression level measured in healthy fibroblasts, whereas deregulation of IL18 and VCAM were significantly (*p* < 0.05) aggravated by Hh inhibition (Figure 5).

**Figure 6:**
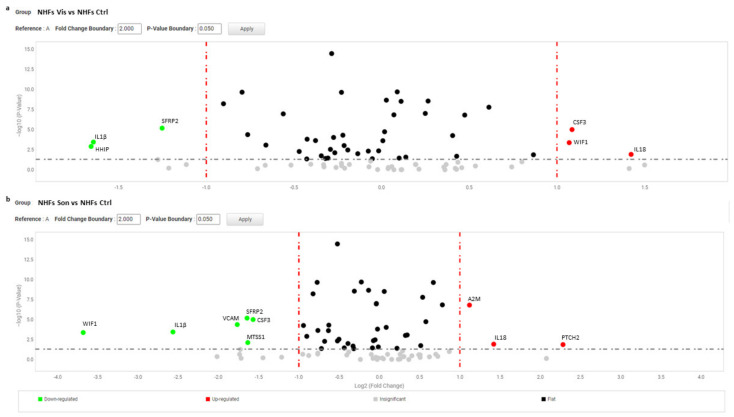
Volcano plot represents gene expression analysis comparing NHFs treated with vismodegib (**a**) and sonidegib (**b**) for 72 h (n = 9) to NHFs untreated (n = 9). (**b**) Analysis was performed using three different housekeeping genes (β-actin, GAPDH and 18S). A one-way ANOVA statistical test with thresholds >2.0 fold-change and *p* < 0.05 defined significant increase are reported in red; significant decrease <0.5 fold-change and *p* < 0.05 reported in green; any fold-difference with *p* ≥ 0.5, e.g., insignificant are reported in grey; ≤2.0 or ≥0.5 difference e.g., flat reported in black.

*HHIP* and *GLI2* gene expression was significantly repressed only in the presence of sonidegib. Notably, in the case of CSF3 we observed divergent regulation in NBCCS-HFs (increase) and CAFs (decrease) (Table 2). The mRNA expression of the major part of inflammatory mediators were not influenced by vismodegib and sonidegib in CAFs, suggesting that in this type of cell this paracrine character is not necessarily driven by Hh signalling. In line with the idea that Hh intracellular signalling is mostly inactive in adult healthy tissue, treatment of normal fibroblasts failed to show any relevant effect with the exception of a IL18 stimulation, a mild but significant decrease of SFRP2 and IL1β with both inhibitors (Figure 6).

Some additional Hh target genes were modified by vismodegib and sonidegib alternatively (Table 2). Finally, the specificity of the effect on mRNA profile observed in the presence of compounds were confirmed by siRNA experiment and following RT-PCR of a sampling of the three most representative mRNA modulated by both molecules (HHIP, IL1β and MTSS1) (Figure S1b and Table 4). Genetic-imposed Hh signalling over-activation represents a quite stable situation that might necessitate a prolonged treatment to achieve prominent repression of SMO activity. Thus, we used a 2-week treatment to simulate the effect of stable Hh inhibition similar to continuative therapeutic regime used in the clinic. Overall, longer treatment impacted firmly on syndromic fibroblast phenotype presenting 10 of the 19 genes responsive to treatments (IL1α, IGF2, IGF1, CCL2, ICAM, MTSS1, MMP1, IL6, SFRP2, and CSF3) with level of expression nearer to normal cells respect to cells treated for 72 h (Table 4 and Supplementary Figure S3).

**Table 4:** Gene expression analysis, 2 weeks.

Gene	NBCCS-HF	NBCCS-HF	NBCCS-HF	NBCCS-HF	NBCCS-HF
		Vis 72 h	Vis 2 weeks	Son 72 h	Son 2 weeks
*IL1α*	252.3	48.1	2.4	23.5	4.0
*HHIP*	195.5	122.9	49.0	42.7	72.3
*IL18*	54.4	10.9	60.7	11.5	1.1
*A2M*	47.1	21.2	13.0	8.4	13.1
*CSF2*	13.6	6.7	7.8	28.4	2.2
*IGF2*	10.6	6.6	1.9	11.8	3.7
*CCL5*	9.3	4.3	0.28	3.6	3.3
*IGF1*	7.3	4.7	0.8	8.0	2.9
*CCL2*	7.1	7.2	2.0	6.9	0.9
*ICAM1*	6.5	4.4	3.5	4.1	2.7
*MTSS1*	6.0	5.5	1.2	3.9	0.28
*HGF*	5.1	2.5	4.2	2.5	2.5
*MMP1*	4.0	2.7	1.4	3.3	1.8
*IL6*	3.7	3.6	1.2	3.3	0.6
*IL1β*	11.5	3.9	8.2	3.7	7.5
*SFRP2*	2.9	7.5	0.6	9.1	0.4
*VCAM1*	2.4	10.1	6.4	6.9	11.9
*CXCL16*	2.4	4.5	2.8	3.7	0.49
*CSF3*	2.2	6.1	0.17	5.5	0.28
*Gli2*	2.1	0.52	1.8	1.9	3.1
*FGF9*	2.0	2.2	1.6	3.0	3.6
*PTCH2*	1.1	1.5	0.2	1.4	1.0
*WIF1*	0.18	0.46	0.12	0.75	0.06
*PTGER3*	0.2	0.57	1.4	0.45	1.3
*CES1*	0.41	1.6	0.8	2.0	0.83
*Gli3*	0.47	0.64	0.65	0.84	0.66
*BDKRB2*	0.47	0.54	0.88	0.63	0.62
*SMO*	1.7	0.77	1.5	0.87	1.7
*Wnt7a*	1.1	1.6	0.43	3.25	0.7
*CASP1*	0.59	0.9	0.56	0.69	0.29
*PTGIS*	1.1	1.7	0.35	1.1	0.71
*CD40*	1.3	1.6	0.82	2.7	0.66
*LTC4S*	0.87	0.62	1.1	2.3	1.6

Gene expression analysis NBCCS-HFs, CAFs compared to NHFs at basal level and following 2 weeks treatment with vismodegib and sonidegib (10 µM). Significantly overexpressed mRNA > 2.0 fold-change and *p* < 0.5 are marked in red, whereas significantly downregulated mRNA < 0.5 fold-change and *p* < 0.5 are marked in blue. The level of mRNAs in healthy fibroblasts was used as reference and arbitrarily indicated as 1.0.

**Figure 7:**
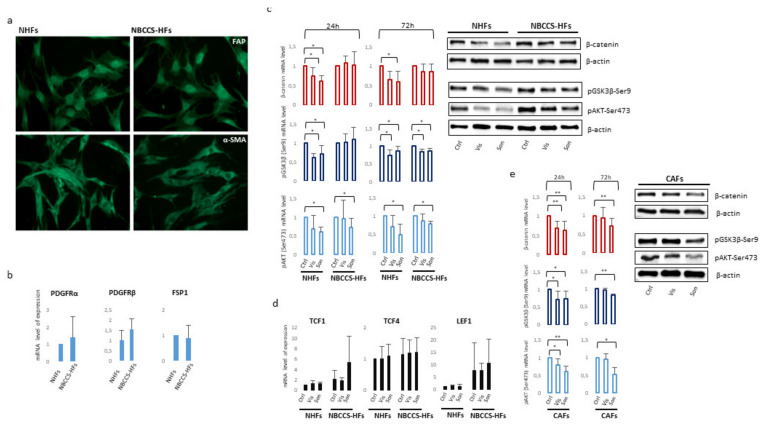
(**a**) Immunofluorescence analysis of α-SMA and FAP protein expression in NBCCS and control cells. Original magnification 20×. (**b**) Semi-quantitative RT-PCR was performed to assess the level of PDGFRα, β and FSP1 mRNA. Histograms report mean ± SD from 9 NBCCS and 9 NHF cell lines. Value from healthy fibroblasts was arbitrarily indicated as 1.0. (**c**) One representative Western blot detecting β-catenin, pSer9-GSK3β and pSer473-AKT of NHFs and NBCCS-HFs. Histograms show densitometric analysis mean ± SD of nine independent experiments. * *p* < 0.05; ** *p* < 0.01. (**d**) Semi-quantitative RT-PCR was performed to assess the mRNA level of expression Wnt-β-catenin pathway transcription factors LEF1, TCF1 and TCF4. Histograms report mean ± SD from 9 NBCCS and 9 NHF cell lines. Value from healthy fibroblasts was arbitrarily indicated as 1.0. (**e**) One representative Western blot detecting β-catenin, pSer9-GSK3β and pSer473-AKT of CAFs. Histograms show densitometric analysis mean ± SD of five independent experiments. * *p* < 0.05; ** *p* < 0.01.

In particular, deregulation of IL6, MTSS1, and MMP1 was completely counteracted after 2 weeks of continuous exposure to SMO antagonists. Notably, vismodegib and sonidegib overturned *SFRP2* and *CSF3* expression only after long treatment whereas, after 72 h treatment, the expression of these two genes resulted extremely high. Long treatment (2 weeks) partially abrogated differences in vismodegib and sonidegib efficacy observed at 72 h. A deeper examination of Table 3 provided additional information on NBCCS-HF and BCC-associated CAF phenotype. Both cell types failed to display a significant different expression of some typical CAF’ markers such as α-smooth muscle actin (α-SMA) and fibroblast activation protein (FAP) at mRNA and protein level suggesting that these genes are not necessarily involved in skin basaloid proliferation promotion by mesenchymal cells (Table 3 and Figure 7a). Similarly, additional evaluation by RT-PCR did not provide evidence of a difference in the level of fibroblast-specific protein 1 (FSP1) and platelet-derived growth factor receptor (PDGFR)-α and β in syndromic fibroblasts (Figure 7b). Consistent with our data, previous transcriptomic analysis of Hh target genes in NBCCS fibroblasts demonstrated no difference in PDGFRα mRNA level compared to healthy cells [49].

Numerous studies have highlighted the importance of molecular crosstalk between the Hh and other signalling cascades, including Wnt pathway. Wnt/β-catenin signalling contributes to the homeostasis of normal skin cells, and its malfunction is implicated in tumour initiation, progression and invasion, but also maintains cancer stem cells which contribute to tumour recurrence [67]. The expression of β-catenin, the key protein regulating Wnt pathway intracellular activity, showed a high heterogenic expression among all fibroblast cell cultures. Altogether, Western blot and densitometric analysis did not evidence statistical difference in the basal level of β-catenin expression in NBCCS-HFs compared to NHFs (data not shown). Vismodegib and sonidegib treatment down-regulates β-catenin at 24 and 72 h in normal fibroblasts whereas NBCCS-HFs resulted less affected especially with the shorter treatment (Figure 7c,d). The mechanism of β-catenin protein reduction did not involve modification of the corresponding mRNA (Table 3), and was associated to an augmented activity of glycogen synthase kinase-3β (GSK3β) since the inactive pSer9-GSK3β was lower in treated cells compared to untreated cells suggesting an accelerated β-catenin ubiquitin-dependent degradation (Figure 7c,d). Correspondingly, pSer473-AKT, an upstream regulator of GSK3β phosphorylation and activity, decreased suggesting the axis pAKT-GSK3β for the control of β-catenin protein turnover and confirming an attenuated effect in the case of syndromic fibroblasts. Again specific interfering of SMO expression confirmed the specificity of down-modulation of the axis pAKT-GSK3β-β-catenin (Figure S1c). Vismodegib and sonidegib treatment exerted a similar effect on CAFs (Figure 7d). Consistent with the synergic regulation of Hh and Wnt pathways, the crosstalk protein SFRP1 carrying a GLI-binding site [68,69] resulted as decreased by SMO antagonists in NBCCS-HF, CAFs and healthy fibroblasts (Table 3). The expression level of β-catenin nuclear partners, the co-transcription factors LEF1, TCF1 and TCF4, did not show any significant variation related to pharmacological treatments (Figure 5). However, a tendency of augmented basal level of expression of LEF/TCF transcription factors was observed in syndromic cells compared to normal cells. The analysis of a wide range of Wnt secreted factors (Wnt5a, Wnt2a, Wnt6, Wnt7a, Wnt9a, Wnt9b, Wnt10b and Wnt11) and members of Dickkopf protein family (DKK1, DKK2 and DKK3) implicated in Wnt/β-catenin signalling miss to identify any specific trait of NBCCS-HFs or variation under Hh inhibitors exposure (Table 2 and Table 3).

### 3.4. Vismodegib and Sonidegib Uptake Revealed Profound Differences

**Figure 8:**
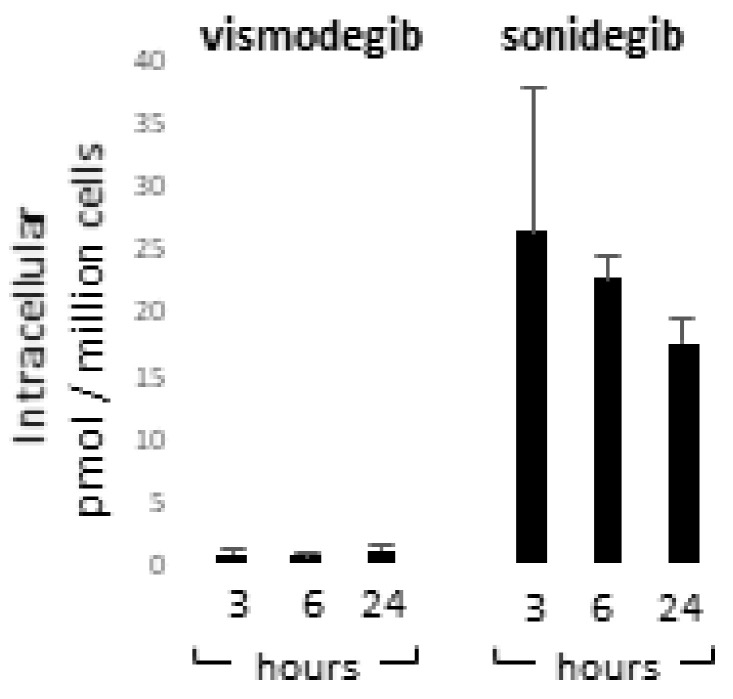
Quantification of vismodegib and sonidegib uptake. Intracellular concentration of vismodegib and sonidegib was evaluated after 3, 6 and 24 h of drugs exposure. Histograms show mean ± SD of three independent experiments.

Vismodegib and sonidegib have been proved to have analogous clinical benefits [36,40]. Herein, disparity in SMO-inhibitory properties observed in vitro, especially the toxic one, represents an unexpected result since these molecules are reported to be similar in terms of IC_50_ for Hh inhibition in human cell-free assays (3.0 µM and 2.5 µM respectively) [70,71]. In GLI-luciferase assay in human embryonic palatal mesenchyme (HEPM) cells, vismodegib inhibited Hh signalling with an IC_50_ of 2.8 nM whereas sonidegib shown an IC_50_ of 12.7 nM [72]. By contrast, in a model using serum-starved human medulloblastoma cells treated with SHH, vismodegib resulted two-fold less potent than sonidegib in restraining GLI1 mRNA production [71]. Moreover, sonidegib displayed similar effectiveness to vismodegib at inhibiting GLI1 and PTCH1 mRNA in in vitro cell cultures and in topically-treated depilated mouse skin [73]. In the same study, SMO-mediated G-protein activation assay and medulloblastoma cells proliferation confirmed similar potency [73]. Thus, overall previous comparative data did not bring out significant inequality in vismodegib and sonidegib effectiveness. Since appreciable off-target effects have been previously excluded at concentrations up to 10 µM [71], we next evaluated the cell internalization of Hh inhibitors by LCMS analysis after 3, 6 and 24 h of drug exposure. At all experimental time points, results evidenced an apparent disparity in the concentration of sonidegib and vismodegib. The sonidegib to vismodegib concentration ratio accounted for 73.3 ± 27.5; 15.1 ± 3.0 and 21.1 ± 1.22 fold-difference at 3, 6 and 24 h respectively (Figure 8). The concentration of vismodegib in the supernatant remained unchanged over time; by contrast, that of sonidegib showed a time-dependent decline opposite to the increasing cell uptake (data not shown).

## 4. Discussion

Antagonistic activity on Hh signalling represents an important therapeutic opportunity for local advanced, metastatic BCCs and for NBCCS patients presenting a dramatic predisposition to develop multiple BCC during their entire life. Despite the motivating use in therapy, in vitro models are rarely reported to study SMO inhibitors since cell lines are not considered to be predictive in the clinic because Hh signalling is mostly silent in adult tissue and the activation of the Hh pathway is not necessarily conserved in pathological cell cultures. In the case of tumour cells, this could be explained by the absence of the microenvironment, specifically fibroblasts that secrete Hh ligands. Moreover, the fact that multiple intracellular signallings susceptible to culture medium composition are implicated in non-canonical Hh pathway activation might complicate data interpretation. In this study, NBCCS-HFs presenting heterozygous germline mutation of *PTCH1* and low passage BCC-CAF cell lines maintained a specific signature ascribable to Hh signalling activity demonstrating the reliability of the model used. Data concerning NBCCS patients confirmed that disease phenotypes may arise from small reductions in PTCH1 activity as in the case of heterozygotic setting presenting one functional copy. All NBCCS-HF cell lines showed a similar Hh signalling signature and comparable sensibility to Hh inhibition (data not shown). The read-out of Hh signalling activation in the oncogenic field is currently in progress. Hence, the definition of its reversion is still being debated. Some groups proposed incremented GLI1 as a reliable marker [74,75,76], whereas others used a panel of multiple genes (two or more), such as *GLI1, PTCH1, HIP, cyclinD1, PTCH2, SFRP1* and *N-myc* to define Hh level of activity [77,78,79]. However, the findings of tumour cells may not be necessarily applicable to normal cells or syndromic fibroblasts carrying a heterozygous loss of function *PTCH1*. In line with this concept, our results showed that the assessment of Hh pathway activity based on *GLI* and *PTCH1* genes expression is not applicable to mesenchymal cells. GLI transcription factors have overlapping as well as distinctive functions. In humans, GLI2 is the primary activator, GLI3 (lacking the C-terminal transactivation domain) is the primary repressor [80,81], and GLI1 is a target gene that acts as an activator in a positive feedback loop [82]. In this study, GLI1 expression resulted as slightly but not significantly increased in untreated NBCCS-HFs and CAFs compared to healthy fibroblasts. Thus, it is possible that moderate overactivity of Hh signalling in non-tumoral cells does not represent a sufficient input to trigger further amplification of the signalling cascade. Upregulation of GLI2 in CAFs and NBCCS-HFs strengthens the proposed idea that GLI2 regulation has a dominant role on the Hh signalling dynamics [83]. At the same time, a lower abundance of GLI3 in the context of endogenous Hh activation endorses a repressor role for this DNA-binding factor. In this study, the most relevant marker of Hh pathway activation is *HHIP* overexpression. *HHIP* is a downstream target gene of Hh signalling responsible for a negative feedback loop due to the capacity to bind and sequester all three Hh ligands away from PTCH receptors [58,84,85,86]. This mechanism probably plays an essential role in moderating the signalling and maintaining a physiological equilibrium in cells of syndromic patients. Accordingly, *HHIP* expression was forcefully modulated by Hh pathway inhibition and SMO siRNA. Additional target genes, such as *CyclinD1*, *PDGFRα*, *VEGF*, *SFRP1*, *Wnt2* were not differentially expressed in syndromic fibroblasts compared to control fibroblasts confirming the distinctive feature of Hh activity in mesenchymal cells. CAFs feature has been documented in several genodermatoses [87]. Previous studies indicated that NBCCS-fibroblasts carrying constitutive activation of Hh intracellular signalling may promote basaloid proliferation by the production of a supportive tumour microenvironment similar to sporadic BCC [50]. Accordingly, the repertoire of growth factors overproduced by syndromic and BCC-associated fibroblasts fully overlaps (IGF1 and 2, HGF, FGF9). Of note, the production of bFGF is lower in syndromic cells compared to normal fibroblasts. A possible explanation for this observation may reside in the evidence that *SMO* and *GLI1* genes are regulated by bFGF [88]. Thus, contraction of bFGF expression could represent a strategy to avoid detrimental excess in Hh signalling cascade. 

A pronounced pro-inflammatory signature is a common character shared by NBCCS-HFs and BCC-CAFs which is consistent with previous studies indicating the Hh pathway as a regulator of cancer immune response [45,89]. However, this aspect seems to be attributable to Hh deregulation only in NBCCS cells, since the expression of the major part of cytokines and interleukins were not perturbed by vismodegib and sonidegib in BCC-stromal fibroblasts. Of note, divergent from other immune response-regulating genes, the expression of CCL2 was rapidly reduced by treatments in CAFs but not in BCC-associated fibroblasts that necessitated prolonged treatment to restore physiological level of this chemokine. Interestingly, the level of CCL2 correlates with the presence of tumour-associated polarized M2 macrophages and poor prognosis in prostate and breast cancer [90,91]. In line with our data, CAFs characterized by the secretion of a high level of pro-inflammatory cytokines are reported to be weakly positive for α-SMA expression [42]. However, this observation seems to be in contrast with a very recent study demonstrating that Hh signalling activity correlates directly to the amount of myofibroblastic CAFs (presenting elevated α-SMA expression) and indirectly to the amount of inflammatory CAFs in pancreatic adenocarcinoma [4].

Another consistent difference between fibroblasts associated with sporadic BCC and syndromic fibroblasts resides in the characteristic over-expression of two Hh signalling components: SMO and PTCH2. Our data are consistent with recent studies in human and mouse models of pancreatic cancer proposing SMO overexpression as a mechanism of activation of this pathway in tumour stroma [48,92]. Of note, even if the global magnitude of Hh target genes deregulation in CAF is lower than in syndromic cells, the efficacy of inhibitors resulted as minor in non-syndromic cells. This is probably related to the fact that targeting CAFs phenotype might be difficult due to the frequent epigenetic permanent modification associated to fibroblast attitude [93,94].

A robust increase of SHH and PTCH2 in normal cells exposed to SMO inhibitors probably aims to restore the minimal basal level of intracellular Hh signalling proving the non-negligible role of minute basic Hh activity in adult cells. However, the overall impact of SMO antagonist treatment was feeble in normal fibroblasts. In line with this idea, data from clinical trials showed that Hh target inhibition in tumours is more pronounced than in the matched normal skin when both tissues were investigated [76]. Opposite deregulated expression of CES1, an enzyme implicated in cellular cholesterol and fatty acids levels [95], might reveal the essential difference of Hh pathway deregulation in NBCCS-HFs and in CAFs, since cholesterol and its oxygenated derivate oxysterols are agonists for SMO [96,97,98] also in absence of Hh ligands [99]. On the contrary, SMO activation is impaired through cholesterol deficiency and sterol depletion [98]. Thus, lipid metabolic abnormalities such as reprogram cholesterol metabolism in a tumor microenvironment may take part in Hh activation. Once again, lipid asset remodelling, in particular lowering the amount of accessible cholesterol (that anti-correlates with PTCH1 activity in normal cells) [100] may act to restrain Hh pathway activity. Additionally, since Hh ligands need to be covalently modified by cholesterol and lipid palmitate to achieve the full activation [101,102], CES-1 modulation might be potentially implicated in limiting SHH bioavailability in NBCCS cells. Vismodegib and sonidegib share essential overlapping Hh inhibitor class-dependent effects. In clinical investigations, these molecules demonstrated similar clinical efficacy, safety, and tolerability profiles [32,103,104]. However, due to the non-coincident study designs, the indirect comparison of pivotal clinical trials used for vismodegib and sonidegib approval (ERIVANCE and BOLT respectively) is enabled and no studies have been reported to evaluate these drugs versus a common comparator [105]. Biological differences between the two Hh inhibitors emerged mostly from pharmacokinetic profiles. First, sonidegib has a longer half-life than vismodegib (29.6 vs. 4–12 days) [106,107]. Both compounds are very highly bound to plasma proteins, but the binding is concentration-dependent for vismodegib [108] and concentration-independent for sonidegib [71,104]. This difference and the major grade of lipophilicity could explain the higher accumulation of sonidegib within the tissue [39,109]. According to clinical data, we observed that the distribution of vismodegib is mainly in the extracellular space whereas sonidegib abundantly penetrates the plasma membrane reaching intracellular concentration about 20-fold higher than vismodegib. The level of intracellular accumulation of SMO inhibitors should in theory be of limited relevance since vismodegib and sonidegib-interacting pocket involves the same SMO-binding cavity that consists in the extracellular stretch of heptahelical membrane domain and a cavity exposed in extracellular space [72,110]. However, our results correlating the intracellular concentration to the efficacy on target genes modulation, strongly suggest that Hh inhibitors may act in the cytoplasm too. A possible explanation is that sonidegib, as reported for two other SMO antagonists, SANT1 and SANT2 [111], blocks the transport of cytoplasmic SMO to the primary cilium of the cell, reducing the level of active SMO. In line with principle, vismodegib been structurally similar to sonidegib might be as well capable to prevent SMO recycling to the membrane but due to limited drug intracellular uptake fails to reach remarkable cytoplasmic concentration. Thus, while inhibiting SMO activity with the well-known canonical mechanism of action, some antagonists might engage cytoplasmic-inactive SMO preventing its primary cilium localization. Therefore, this represents a second mechanism by which inhibitors can attenuate SMO activity potentiating the pharmacological effectiveness. An augmented pharmacological effect for sonidegib, due to the secondary intracellular interaction with SMO, may explain the powerful effect at 72 h of treatment that is partially confirmed after 2 weeks of drug exposure. This augmented efficacy might be useful in therapy to reconsider active doses, reduce side effects, and enlarge the possible spectrum of target patients. Although vismodegib and sonidegib have only been approved for cases of advanced BCCs, the question has been raised whether they would be useful for the greater majority of BCCs that present the typical of-fice setting (i.e., non-advanced). Moreover, Hh inhibitors might be used as neoadjuvant therapy to decrease primary tumour size before excision reducing the complexity and extent of closure [112].

## 5. Conclusions

In conclusion, in this study we provided evidence that targeting the Hh pathway impacting on fibroblast tumour supportive functions might be considered a therapeutic option for BCC independent of the mutation status of Hh components in tumour cells. The advantage of targeting stromal cells in addition to neoplastic cells resides in a more durable response since normal cells have a relatively stable genetic constitution compared to cancer cells that easily develop secondary resistance to target therapies. However, further investigations will address the question of how CAF targeting could be improved. Also the evidence that a cancer-prone phenotype of NBCCS fibroblasts was significantly repressed by SMO inhibitors strengthens the use of this class of compound in the management of syndromic patients.

## Data Availability

The datasets used or analysed during the current study are available from the corresponding author on reasonable request.

## Supplementary Materials

The following are available online: https://www.mdpi.com/article/10.3390/cancers13225858/s1, Figures S1: (a) MTT assay of NBCCS-HFs, CAFs and NHFs transfected with specific SMO siRNA for 72 h. Data represent the average of experiments performed with 2 different SMO siRNA. (b) Semi-quantitative RT-PCR was performed to assess the level of SMO, IL1β, MTSS1 and HHIP level of expression in cells transfected with SMO siRNA (72 h). Histograms report mean ± SD of 2 set of experiments performed with 2 different SMO siRNA. (c) One representative western blot detecting β-catenin, pSer9-GSK3β and pSer473-AKT after 72 h treatment with siRNA (NS siRNA or SMO siRNA); Figure S2: Volcano plot represents gene expression analysis comparing NBCCS HFs treated with vismodegib (a) and sonidegib (b) for 2 weeks (n = 9). (b) Analysis was performed using three different housekeeping genes (β actin, GAPDH and 18S). One way ANOVA statistical test with thresholds >2.0 fold change and *p* < 0.05 defined significant increase are reported in red; significant decrease <0.5 fold change and *p* < 0.05 reported in green; any fold difference with *p*≥ 0.5, e.g., insignificant are reported in grey; ≤2.0 or ≥0.5 difference e.g., flat reported in black; Table S1: Healthy donors characteristics; Table S2: BCC patients characteristics; Table S3: Primers for Real Time Quantitative Polymerase Chain Reaction.
