# Supplementary material for: Evaluation of Hedgehog Pathway Inhibition on Nevoid Basal Cell Carcinoma Syndrome Fibroblasts and Basal Cell Carcinoma-Associated Fibroblasts: Are Vismodegib and Sonidegib Useful to Target Cancer-Prone Fibroblasts?
Source: Cancers (Basel). 2021 Nov 22;13(22):5858. doi: 10.3390/cancers13225858 (PMC8616531; doi:10.3390/cancers13225858)
Supplement: Supplementary file 1 [file cancers-13-05858-s001.zip › cancers-1439705-supplementary.pdf]

Table S1

Healthy donors characteristics

Donors	Age	Sex	Skin biopsy location
**NHF-Ad17**	93	M	Abdomen
**NHF-Ad19**	60	M	Abdomen
**NHF-Br15**	84	F	Arm
**NHF-D16**	36	M	Dorsum
**NHF-D24**	40	M	Dorsum
**NHF-D27**	39	M	Dorsum
**NHF-G9**	63	F	Leg
**NHF-G13**	77	M	Leg
**NHF-Ing5**	96	M	Inguinal
**NHF-Pa6**	55	F	Occipital area
**NHF-S16**	62	F	Breast
**NHF-Sp2**	29	M	Schiuder

Table S2

BCC patients characteristics

Patients	Age	Sex	Skin biopsy location
**BCC-233**	78	M	Leg
**BCC-748SF**	78	F	Scalp
**BCC-800AN**	87	F	Dorsum
**BCC-1000NA**	67	F	Dorsum
**BCC-1PE**	66	M	Abdomen
**BCC-2TW**	75	M	Neck
**BCC-4CS**	70	M	Dorsum

Table S3

Primers for Real Time Quantitative Polymerase Chain Reaction

Target Gene	Foward Primer	Reverse primer
β-Actin	5'-GACAGGATGCAGAAGGAGATTACT-3'	5'-TGATCCACATCTGCTGGAAGGT-3'
TCF1	5'-CCA AGA ATC CAC CAC AGG AGG-3'	5'-GCA GCC TAG AGC ACT GTC AT-3'
TCF4	5'-AAG ACT GGA TGA TGC TAT TC-3'	5'-CTG TTG GCT GAA AGA AGG-3'
Lef1	5'-TTC CTT GGT GAA CGA GTC-3'	5'-GGG TCC CTT GTT GTA GAG-3'
PDGFRα	5'-GTG TGG GAC ATT CAT TGC GG-3'	5'-TAG GCT CAG CCC TGT GAG AA-3'
PDGFRβ	5'-GGA GAG GGC AGT AAG GAG GA-3'	5'-GAA GCC GCA TGG TGT CCT TG-3'
FSP1	5'-CTT GGT TTG GTG CTT CTG AGA-3'	5'-TGG ACA CCA TCA CAT CCA GG-3'

Fig. S1

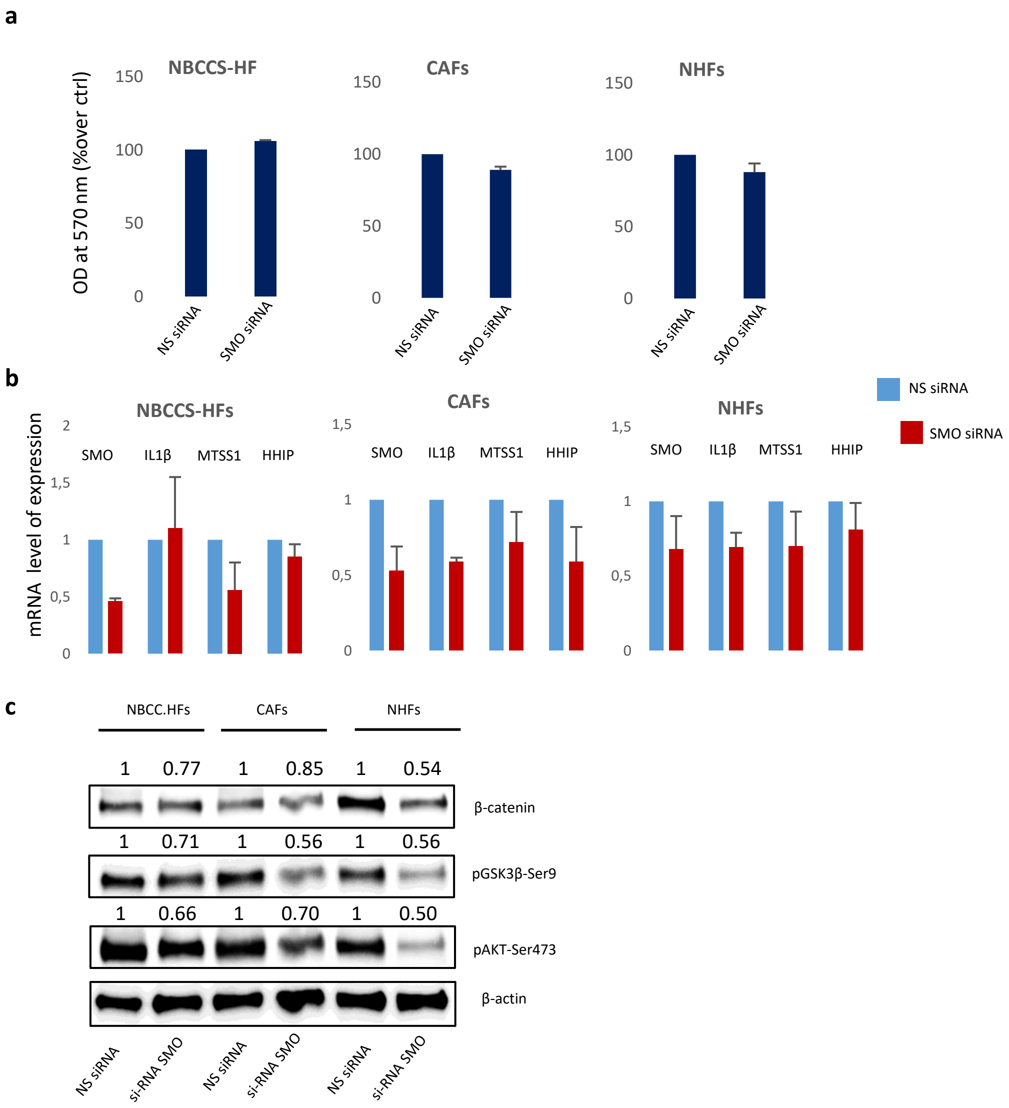

Table S4 Gene expression analysis

Gene	NBCCS-HF NS-siRNA	NBCCS-HF SMO-siRNA	CAF NS-siRNA	CAF SMO-siRNA	NHF NS-siRNA	NHF SMO-siRNA
SMO	4.9±5.4	**2.2±0.11**	9.6±9.5	**5.1±1.5**	1.0	**0.68±0.22**
IL1β	6457.7±9666.3	7102.3±2905.3	30.3±23.5	**17.9±5.0**	1.0	**0.69±0.10**
MTSS1	7.5±3.4	**4.2±1,79**	4.73±3.4	**3.40±0.9**	1.0	**0.70±0.23**
HHIP	873.3±1192.4	**733.6±87.3**	38.3±37.0	**22.8±8.9**	1.0	**0.81±0.18**

Figure S1: (a) MTT assay of NBCCS-HFs, CAFs and NHFs transfected with specific SMO siRNA for 72h. Data represent the average of experiments performed with 2 different SMO siRNA. (b) Semi-quantitative RT-PCR was performed to assess the level of SMO, IL1β, MTSS1 and HHIP level of expression in cells transfected with SMO siRNA (72h). Histograms report mean ± SD of 2 set of experiments performed with 2 different SMO siRNA. (c) One representative western blot detecting β-catenin, pSer9-GSK3β and pSer473-AKT after 72h treatment with siRNA (NS siRNA or SMO siRNA).

Fig. S2

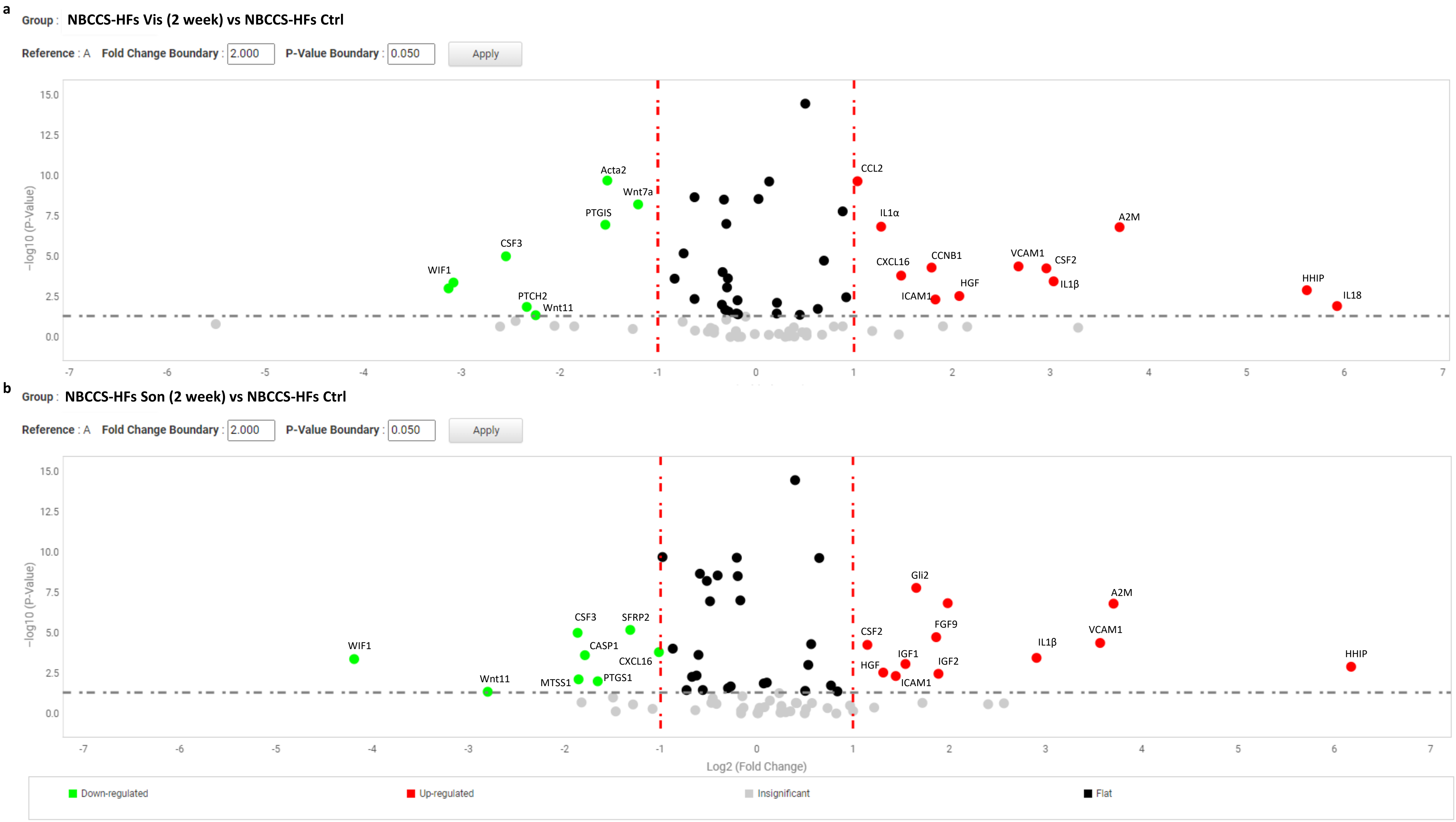
a
Group : NBCCS-HFs Vis (2 week) vs NBCCS-HFs Ctrl
Reference : A Fold Change Boundary : 2.000 P-Value Boundary : 0.050 Apply
-log10 (P-Value)
15.0
12.5
10.0
7.5
5.0
2.5
0.0
-7 -6 -5 -4 -3 -2 -1 0 1 2 3 4 5 6 7
Acta2
Wnt7a
PTGIS
CSF3
WIF1
PTCH2
Wnt11
CCL2
IL1α
A2M
CXCL16
CCNB1
VCAM1
CSF2
IL1β
HGF
ICAM1
HHIP
IL18
b
Group : NBCCS-HFs Son (2 week) vs NBCCS-HFs Ctrl
Reference : A Fold Change Boundary : 2.000 P-Value Boundary : 0.050 Apply
-log10 (P-Value)
15.0
12.5
10.0
7.5
5.0
2.5
0.0
-7 -6 -5 -4 -3 -2 -1 0 1 2 3 4 5 6 7
Log2 (Fold Change)
CSF3
SFRP2
WIF1
CASP1
CXCL16
Wnt11
MTSS1
PTGS1
Gli2
A2M
FGF9
CSF2
VCAM1
IL1β
IGF1
HGF
IGF2
ICAM1
HHIP
Down-regulated
Up-regulated
Insignificant
Flat

Figure S2: Volcano plot represents gene expression analysis comparing NBCCS-HFs treated with vismodegib (a) and sonidegib (b) for 2 weeks (n=9). (b) Analysis was performed using three different housekeeping genes (β-actin, GAPDH and 18S). One-way ANOVA statistical test with thresholds >2.0 fold-change and p <0.05 defined significant increase are reported in red; significant decrease <0.5 fold-change and p<0.05 reported in green; any fold-difference with p≥0.5, *e.g.* insignificant are reported in grey; ≤2.0 or ≥0.5 difference *e.g.* flat reported in black.
